# Metastatic Tumors of the Sinonasal Cavity: A 15-Year Review of 17 Cases

**DOI:** 10.3390/jcm8040539

**Published:** 2019-04-19

**Authors:** Miao-Hsu Chang, Ying-Ju Kuo, Ching-Yin Ho, Edward C. Kuan, Ming-Ying Lan

**Affiliations:** 1 Department of Otorhinolaryngology-Head and Neck Surgery, Taipei Veterans General Hospital, Taipei 11217, Taiwan; maiohsu@gmail.com; 2 Department of Pathology, Taipei Veterans General Hospital, Taipei 11217, Taiwan; yjkuo2@vghtpe.gov.tw; 3 School of Medicine, National Yang-Ming University, Taipei 11221, Taiwan; tigertpe@gmail.com; 4 Department of Otolaryngology, Cheng Hsin Hospital, Taipei 11220, Taiwan; 5 Department of Otolaryngology-Head and Neck Surgery, University of California, Irvine, Orange, CA 92868, USA; eckuan@hs.uci.edu

**Keywords:** sinonasal malignancy, metastases, paranasal sinuses, cancer, maxillary sinus

## Abstract

Extranasal cancers that metastasize to the sinonasal cavity are very rare. To date, there are only limited reports regarding this rare condition within the literature. Therefore, we retrospectively reviewed all patients diagnosed with metastatic cancer of the sinonasal tract from 2003 to 2018 at a tertiary academic medical center. Patient demographic data, clinical presentation, treatment modalities, and outcomes were investigated. There were a total of 17 patients (9 males and 8 females) included in the analysis. The mean age was 56.8 years (range 27–80). The most common primary malignancies were hepatocellular carcinoma (*n* = 3) and gastrointestinal tract adenocarcinoma (*n* = 3). The most common site of metastasis was the nasal cavity (*n* = 8). Five patients received radical tumor resection and the others underwent radiotherapy, chemotherapy, or combined chemoradiotherapy. The 2-year survival was 28%. In summary, metastasis to the sinonasal cavity remains extremely rare. A high degree of suspicion regarding the possibility of metastatic spread to the sinonasal region is necessary for patients with a previous history of malignancy who present with new sinonasal symptoms. The treatment strategy of sinonasal metastatic cancer is usually palliative therapy and the prognosis remains poor. However, early detection and diagnosis, coupled with aggressive treatment, may improve patient quality of life.

## 1. Introduction

Sinonasal malignancies are extremely rare and generally present as primary malignancies. They account for approximately 3% of upper respiratory tract malignancies [1,2,3,4]. The incidence of metastatic tumors in the sinonasal cavity is even more unlikely [5,6,7,8]. The most common origins of metastatic sinonasal malignancy are the kidneys, lungs, urogenital ridge, breasts, gastrointestinal tract, and thyroid [5,9]. In the literature, renal cell carcinoma (RCC) is the most common metastatic cancer of the sinonasal tract, accounting for almost half of all cases. Other common metastatic cancers are bronchogenic carcinoma and urogenital ridge malignancies, such as prostate cancer and female urogenital cancer [10,11]. 

Due to its limited occurrence, information regarding sinonasal metastases remains to be investigated. In the current study, we present tumor clinicopathologic characteristics, treatment modalities, and prognosis of 17 patients diagnosed with sinonasal metastases.

## 2. Materials and Methods

### 2.1. Patients

This was a retrospective review of patients diagnosed with sinonasal metastases and treated at Taipei General Veterans Hospital in Taiwan between 1 January 2003 and 31 December 2018. Institutional Review Board approval was obtained. Data regarding patient demographics, presenting signs and symptoms, tumor pathology, primary tumor site, metastatic sites, laboratory studies, relevant imaging scans, treatment modalities, complications, and outcomes were collected.

### 2.2. Statistical analysis

Quantitative data were summarized as the mean ± standard deviation (SD) and categorical variables as percentages. IBM SPSS 20.0 software (IBM Corp., Armonk, NY, USA) was used for the statistic analysis. The Kaplan–Meier method was used to calculate survival trends.

## 3. Results

### 3.1. Patients

Demographic data are summarized in Table 1. There were 17 patients identified during the study period, including 9 males and 8 females. Age at diagnosis ranged from 27 to 80 years (mean 56.8 ± 14.2 years). In three cases, the primary and metastatic lesions were found synchronously, and the other 14 cases experienced metastasis after the primary tumors were treated. Time from treatment to the development of metastases ranged from 2 months to 18 years and 7 months.

**Figure 1:**
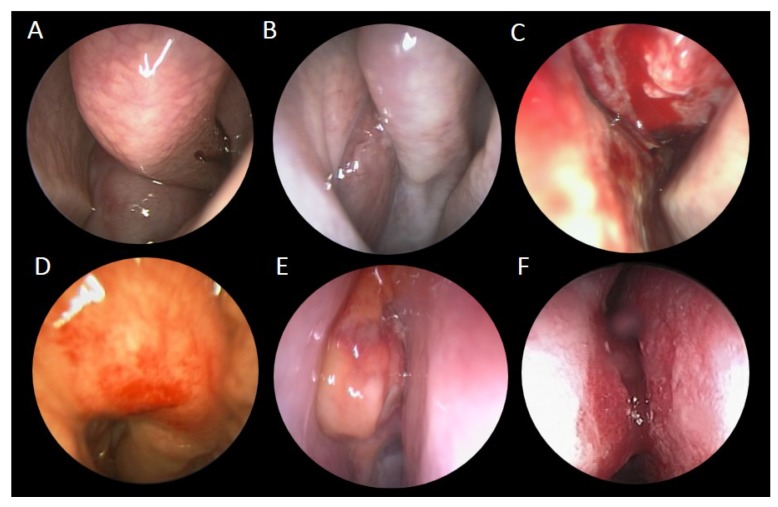
Endoscopic views of various sinonasal metastatic cancers. Endoscopic examinations showed various metastatic sinonasal malignancies, which usually appeared as reddish, fragile, and hemorrhagic masses in the nasal cavity or sinuses: (**A**) retroperitoneum leiomyosarcoma, (**B**) thyroid poorly differentiated carcinoma, (**C**) breast invasive ductal carcinoma, (**D**) rectal adenocarcinoma, (**E**) hepatic cell carcinoma, and (**F**) lung adenocarcinoma.

**Figure 2:**
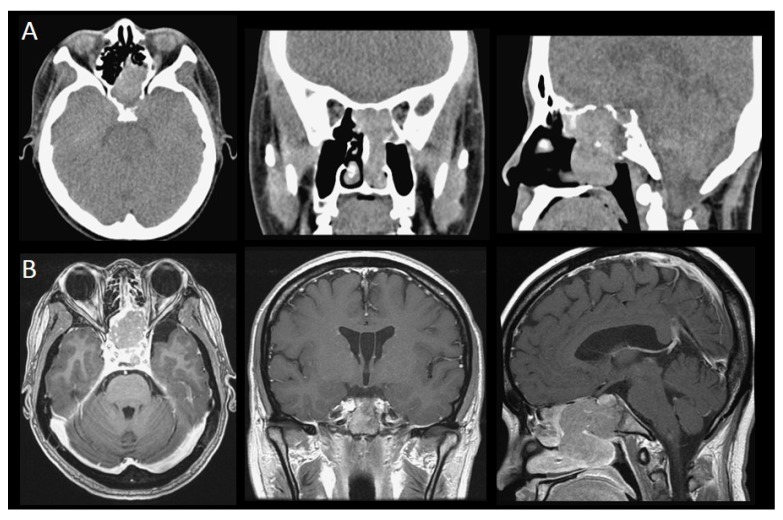
CT and MRI images of sinonasal metastatic cancers: (**A**) A 37-year-old female diagnosed with metastatic retroperitoneum leiomyosarcoma. The CT scan revealed that the tumor involved the nasal chamber, bilateral sphenoid sinus, left side of the posterior ethmoid sinus, left aspect of the sellar floor, and the clivus. (**B**) A 45-year-old female diagnosed with retrorectal neuroendocrine carcinoma. The MRI scans revealed that the tumor involved the sphenoid sinus, sella, suprasella, left cavernous sinus, and pituitary gland.

In our study, there were eight unilateral and seven bilateral sinonasal metastases. As for metastatic sites, eight cases metastasized to the nasal cavity, six cases to the skull base, five cases to the nasal septum, four cases to the maxillary sinus, four cases to the ethmoid sinus, four cases to the sphenoid sinus, and one case to the frontal sinus. Among them, nine cases manifested as a single lesion, while seven cases were multifocal lesions. Common symptoms included epistaxis (*n* = 7), headache (*n* = 3), nasal obstruction (*n* = 2), diplopia (*n* = 1), extraocular movement limitation (*n* = 1), and facial swelling (*n* = 1) (Table 1). For cases with metastatic lesions involving the nasal cavity, endoscopy often revealed hypervascularity, friable, or polypoid lesions, which bled easily after touch (Figure 1). CT or MRI were commonly utilized to evaluate the extent of tumor invasion (Figure 2).

**Figure 3:**
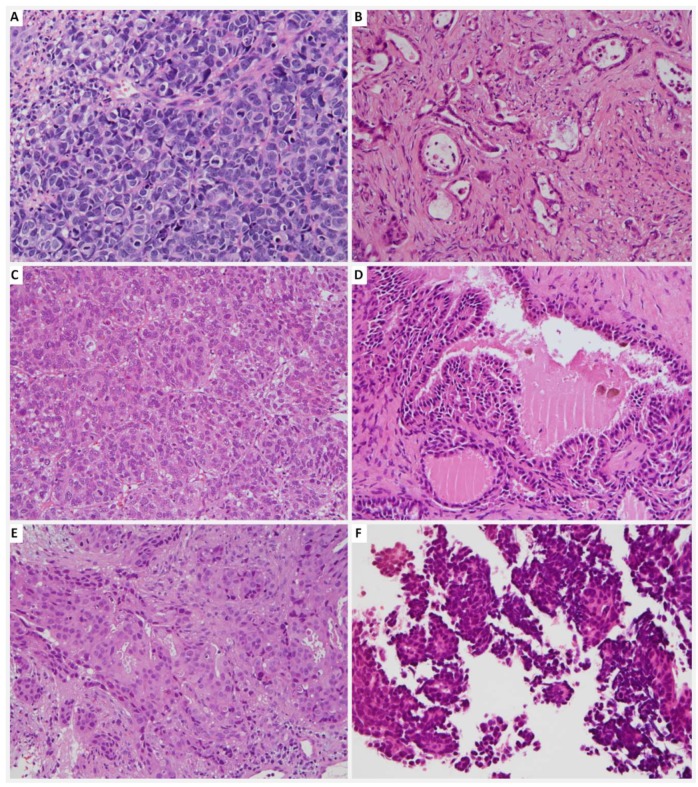
Pathologic images of various sinonasal metastatic cancers: (**A**) Metastatic neuroendocrine carcinoma; (**B**) Metastatic colorectal adenocarcinoma of the colon; (**C**) Metastatic hepatocellular carcinoma; (**D**) Metastatic papillary thyroid carcinoma; (**E**) Metastatic breast carcinoma; and (**F**) Metastatic pulmonary adenocarcinoma.

Pathology of the primary tumors were diverse, including hepatocellular carcinoma (HCC) (*n* = 3), gastrointestinal tract adenocarcinoma (*n* = 3), invasive ductal carcinoma of the breast (*n* = 2), retroperitoneal leiomyosarcoma (*n* = 2), poorly differentiated thyroid carcinoma (*n* = 1), thyroid papillary carcinoma (*n* = 1), melanoma of the foot (*n* = 1), osteosarcoma of the tibia (*n* = 1), laryngeal mucoepidermoid carcinoma (*n* = 1), retrorectal neuroendocrine carcinoma (*n* = 1), and lung adenocarcinoma (*n* = 1) (Table 2) (Figure 3). There were no cases of renal cell carcinoma.

### 3.2. Treatment Modality

There were five patients who underwent radical tumor excision, and one who underwent tumor debulking. Five patients received radiotherapy, and 10 patients underwent palliative chemotherapy (Table 3).

### 3.3. Survival

**Figure 4:**
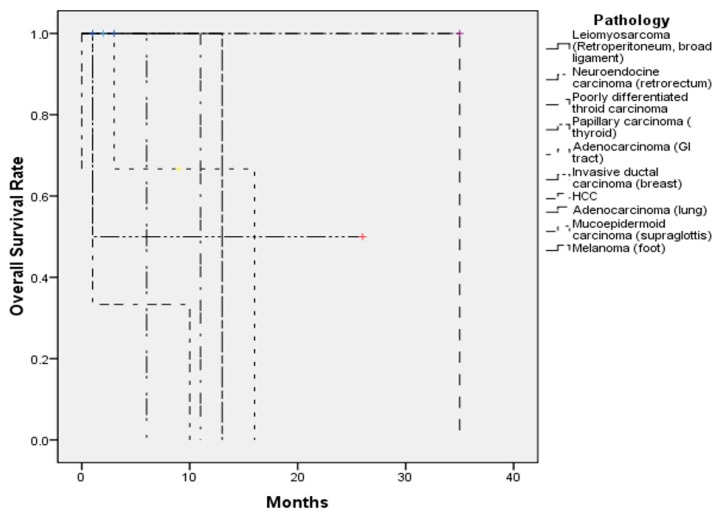
The overall survival of the 17 patients with sinonasal metastases in our study.

The follow-up duration ranged from 1 month to 35 months. One patient was lost to follow-up (case No. 1). At the last follow-up, three patients were free from disease (cases 2, 5, and 16), four patients were still receiving regular follow-ups or treatment for residual and/or recurrent disease (cases 4, 8, 11, and 12), and nine patients died from disease (cases 3, 6, 7, 9, 10, 13, 14, 15, and 17) (Table 3). The 1-year survival was 47%, and the 2-year survival was 28% (Figure 4).

## 4. Discussion

Sinonasal metastases from distant extranasal sites is exceedingly rare. In the literature, nearly half of sinonasal metastases originated from the kidneys, followed by the lungs, breasts, colon, prostate, urogenital ridge, gastrointestinal tract, thyroid, and pancreas (Table 4) [1,5,9,12,13,14,15,16,17,18,19]. In our study, there were four sinonasal metastatic cases originating from the stomach and three cases from the liver, making gastrointestinal adenocarcinoma and hepatocellular carcinoma the top two common primary tumors. Previous cases have been largely reported in the Western literature [5,6,20,21,22,23,24,25]. However, the incidence and distribution of malignant tumors might be diverse between different geographic areas due to distinct genetic or environmental factors [26]. Among sinonasal metastases, the incidence of prostate cancer and breast cancer ranked first and second highest in North America and Europe, respectively. In North America, the incidence of kidney cancer ranked after colon cancer, melanoma, and thyroid cancer. However, in Europe, the incidence of RCC is higher than that of melanoma. In East Asia, the incidence of cancers of the respiratory tract have been reported to be the highest, followed by stomach, liver, breast, and colorectal cancer; in contrast, the incidence of kidney cancer was relevantly low [27]. According to the Taiwan Food and Drug Administration (TFDA) statistics and a study investigating cancers between 2002 and 2012, the most common malignancy overall in Taiwan was also breast cancer, followed by colorectal, respiratory tract, and liver cancers [28]. Huang et al. proposed that the differences in primary cancers leading to metastatic sinonasal cancers compared to that in Western countries might be due to a unique cancer epidemiology exclusive to Taiwan [26].

It is well-established that chronic hepatitis B virus (HBV) and hepatitis C virus (HCV) infections are important risk factors for the development of HCC [29,30]. Incidence of HBV and HCV infection was high in Taiwan before the launch of the HBV vaccine and other means of hepatitis virus control, which explains the relatively higher incidence of HCC in Taiwan [13,31,32]. Therefore, HCC was identified as one of the most common sinonasal metastases in our study, which is different from Western studies. 

Prior reports have identified the maxillary sinus as the most common site of metastasis, followed by the sphenoid, ethmoid, frontal sinuses, and, finally, the nasal cavity [5,9,11,18,33,34,35]. However, in our study, the nasal cavity was the most common site for metastasis. This is different from previous studies and may suggest different biological behavior for distant metastatic spread based on different primaries.

The symptoms for such tumors are usually non-specific, similar to that of primary sinonasal tumors [36], and are related to the characteristics of the primary tumor and the metastatic sites [37,38]. Due to the fact that RCC is often highly vascular [39], metastasis from RCC to the sinonasal tract is usually accompanied by epistaxis. A tumor affecting the nasal cavity may present with epistaxis, increased nasal secretions, and nasal obstruction. Maxillary sinus tumors may present with symptoms of facial swelling, facial numbness, or eye symptoms. Ethmoid and sphenoid sinus tumors may result in headache, proptosis, diplopia, or vision changes [5,20,33]. Horner’s syndrome has been reported to result from lateral sphenoid sinus erosion or infratemporal fossa lesion [12]. If the tumor involves the cavernous sinus or the skull base, patients may present with various cranial nerve palsies, such as diplopia, ptosis, or visual impairment [13,35,40]. In our study, the most common symptom was epistaxis, followed by headache and nasal obstruction. For those with frequent epistaxis and severe nasal obstruction, surgery helps alleviate the symptoms, which might improve quality of life. 

Time from treatment to the development of metastases ranged from 2 months to 18 years and 7 months. The wide variation may be related to the initial M (the presence of metastasis) stage of the patient when diagnosed with the cancer. For cases with initial presentation of other metastatic sites, which suggests widespread disease, the duration of the development of sinonasal metastasis should be shorter. However, in our study, the mean metastatic duration for an initial M0 stage was 22 months (4 cases), which was shorter compared to patients with an initial M1 stage (25 months, 8 cases), with no significant difference between the two groups (*p* > 0.05). Tumor biology may play a major role in metastatic duration, though the small sample size of this study limits more in-depth analysis.

Hematogenous [5,11,41,42,43,44] and lymphatic spread [13,37,45] are two different pathways of metastasis. For hematogenous travel, tumor cells have to undergo intravasation, which is the process of passing through endothelial cells into the blood vessels. Tumor cells circulate via the valveless vertebral venous (Batson’s) plexus and can enter the pterygoid plexus and cavernous sinus, as well as the nasal and paranasal sinuses [9,41,46,47]. As for lymphatic spread, the tumor emboli flow into regional lymphatic channels and drain to the thoracic duct, and then flow in retrograde through the intercostal, mediastinal, or supraclavicular lymph vessels to the head and neck region [9,13,37]. Whether tumor cells metastasize via hematogenous or lymphogenous pathways depends on multiple factors, including the density of vascularization around the tumor or the secondary site, the biologic behavior of the primary tumor, and the presence or absence of lymph node involvement [48]. 

Though rare, it is critical to keep sinonasal metastases within the differential diagnoses for sinonasal masses. Identification of the origin of the metastatic sinonasal cancer differs from case to case. In general, histopathologic analysis will be required to make the diagnosis. It would be prudent to obtain imaging (CT and/or MRI) ahead of time to rule out intracranial extension, as well as to determine the vascularity of the lesion, and also for pre-operative surgical evaluation. For extranasal primary cancers, expression of specific biomarkers, such as carcinoembryonic antigen (CEA) and prostate-specific antigen (PSA), may be considered. For instance, if the patient had a history of breast cancer, the expression of human epidermal growth factor receptor 2 (HER2) and glandular morphology in malignant cells should be checked. 

Primary sinonasal malignancies and sinonasal metastases may overlap in clinical behavior and histopathologic appearance. Three of our cases were metastatic adenocarcinomas of gastrointestinal or colorectal origins. This is to be distinguished from primary sinonasal intestinal-type adenocarcinoma, which may share similar morphology and immunophenotyping, such as immunoreactivity for CK20 and cdx2. In our study, there were also two metastatic breast carcinomas, of which the morphology may be variable and may lack specificity. Therefore, the lesion may not be easily separated from a high-grade non-intestinal type adenocarcinoma. One of our patients was a metastatic rectal neuroendocrine carcinoma, and its pathologic findings were identical to that of a primary sinonasal neuroendocrine carcinoma. Therefore, the precise diagnosis of sinonasal metastases relies on the combination of clinical, radiological, and pathologic information. 

Metastases to the sinonasal cavity represent advanced distant disease and are naturally associated with poor prognosis, and, in the vast majority of cases, may not be treated with a curative intent. Mickel and Zimmerman reviewed the survival in 26 cases of malignancies that metastasized to the sphenoid sinus. Only nine cases lived for more than six months and four cases survived for at least two years [12]. Multifocal metastases in the sinonasal region have been noted to be much more common than a single metastasis [21]. In our study, there were seven cases with multifocal metastatic tumor in the sinonasal region, and nine cases with a single lesion. The location and number of metastatic lesions mainly determine whether the lesion may be operable or not. Multiple metastatic lesions and proximity to vital structures increase the challenges of surgical resection for palliation, which would be the only possible goal in these cases [37]. In many cases, resections may be approached endoscopically. For lesions that cannot always be excised radically, other multimodality treatments, such as radiotherapy, chemotherapy, hormone therapy for urogenital cancer, or iodine ablation therapy for thyroid cancer, may be considered, and should be chosen based on the characteristics of the primary tumor. One consideration for palliative surgical candidates is preoperative embolization of feeding vessels to reduce intraoperative bleeding [9]. Most sinonasal tumors receive blood supply from external carotid artery branches, which would be amenable to embolization [7,9,12]. In our opinion, whether or not patients receive surgery depends on several factors, including the general health condition of the patient, the preferences of the surgeon, and tumor biology. We would recommend surgery if the tumor caused frequent nasal bleeding or severe nasal obstruction (to improve quality of life), and if the patient could tolerate general anesthesia. The location and size of the metastatic tumor should be readily accessible. Classically, surgery is not routinely considered for metastatic disease. However, surgery may provide some chance for these patients to live longer (decreasing tumor burden for later chemotherapy control), and to also achieve a better quality of life (alleviating symptoms of nasal bleeding and nasal obstruction).

In our study, 10 (58.8%) of the 17 patients died in the follow-up period. The shortest survival time was 2 months and the longest was 35 months. There were three patients alive without obvious residual sinonasal metastatic lesions, and three patients alive with either residual sinonasal metastases or other distant metastases. The 1-year survival was 47%, and the 2-year survival was 28.1%. There are studies that showed that differences in prognosis are apparent based on the presence of isolated or widespread disseminated disease [7], with longer survival noted for an isolated sinonasal metastasis [22]. In addition, slightly higher survival has been noted in patients that receive surgery than those who receive radiotherapy alone [7]. However, in our study, there was no significant difference in survival whether patients received surgery, whether the sinonasal lesions were multifocal, or whether there was isolated or widespread disseminated disease (Supplementary Figure S1). The insignificant findings may be related to the small number of cases, which is harder for meaningful statistical analysis. 

Indeed, pathology may play a major role in patient survival. All patients with HCC and melanoma died of the disease, which is characteristic of the known poor survival when compared to other tumor types. One case with thyroid papillary carcinoma, a malignancy with a good prognosis, received surgical excision of a nasal septal metastatic lesion and was still alive without obvious recurrence. In regard to targeted therapy for metastatic breast cancer, one patient receiving target therapy was still alive, while another who did not receive targeted therapy died of the disease. Four out of seven patients receiving target therapy died regardless of the cancer type, including one case of HCC, one case of lung adenocarcinoma, one case of gastric adenocarcinoma, and one case of cecal adenocarcinoma. Since this was a retrospective study, there may be some bias to stratify the patients based on receiving targeted therapy, due to its recent development. Cancer cases in more recent years would be more likely to receive the novel targeted therapies compared to those from prior years. In our study, 11 patients had widespread metastatic disease when sinonasal metastasis (e.g., lung, bone, liver, brain) was diagnosed, while 5 cases presented with isolated sinonasal metastasis. 

In our study, gastrointestinal adenocarcinoma and hepatocellular carcinoma were the top two common primary tumors that metastasized to the sinonasal tract, which is quite different from reports on Western countries. The nasal cavity was the most common site for metastasis, which is also quite different from previous reports where the maxillary sinus was the most common site. The similar findings included the mean age, the sex distribution, and the poor prognosis (Table 4). There are several limitations to our study. First, it is a retrospective study, which makes complete data collection and verification difficult. Second, the small number of cases for such a rare condition, may prevent meaningful statistical analysis. Finally, treatment plans for each patient may be impacted by physician and/or patient preference, where electing to be more aggressive with treatment may affect survival.

## 5. Conclusions

Sinonasal metastases from extranasal primary malignancies are rare, and patients usually suffer from non-specific symptoms. Treatment modalities differ based on the origin and characteristics of the primary tumor. The prognosis remains poor, though early detection and aggressive treatment may help to improve quality of life.

## Figures and Tables

**Table 1:** The demographic and clinical characteristics of the patients and the tumors.

	Case Number (*n* = 17)
**Age**	27–80 (mean 56.8 ± 14.2)
**Sex**	M:F = 9:8
**Metastatic time**	2 months–18 years and 7 months
**Symptoms**	
Epistaxis	7
Headache	3
Nasal obstruction	2
Diplopia	1
Extraocular movement limitation	1
Facial swelling	1
**Side**	
Unilateral (L/R)	8 (3/5)
Bilateral	7
**Sinonasal metastases**	
Single metastases	9
Multifocal metastases	7
**Widespread metastasis**	11
**Sinonasal metastatic sites**	
Nasal cavity	8
Skull base	6
Nasal septum	5
Maxillary sinus	4
Ethmoid sinus	4
Sphenoid sinus	4
Frontal sinus	1

**Table 2:** Primary tumor origins and pathologies.

	Number (*n* = 17)
**Primary tumor site**	
-GI tract	4
-Liver	3
-Breast	2
-Thyroid	2
-Retroperitoneum	1
-Broad ligament	1
-Supraglottis	1
-Lung	1
-Tibia	1
-Foot	1
**Pathology**	
-Adenocarcinoma (GI tract)	3
-Hepatocellular carcinoma (HCC)	3
-Invasive ductal carcinoma (breast)	2
-Leiomyosarcoma (retroperitoneum, broad ligament)	2
-Poorly differentiated carcinoma (thyroid)	1
-Papillary carcinoma (thyroid)	1
-Adenocarcinoma (lung)	1
-Neuroendocrine carcinoma (retrorectum)	1
-Mucoepidermoid carcinoma (supraglottis)	1
-Osteosarcoma (tibia)	1
-Melanoma (foot)	1

**Table 3:** Summary of the cases of sinonasal metastatic cancer in our study.

No.	Age/Sex	Symptoms	Primary Tumor	Clinical Stage before Metastasis to Sinonasal Region	Sinonasal Metastatic Site	Time Before Metastasis	Extrasinonasal Metastasis (Initial)	Extrasinonasal Metastasis (Before Sinonasal Metastasis)	Treatment Modality	Follow-up Duration	Disease Status
1	27M	Headache Ptosis	Tibiaosteosarcoma	Stage III (M1)	NA	4y5m	N	Lung	CT (Ifosfamide + Etoposide)	NA	NA
2	37F	Epistaxis	Retroperitoneum leiomyosarcoma	Stage IV (M1)	Sphenoid, ethmoid sinuses, clivus	3y4m	N	Liver	Tumor resection + RT	1m	Alive, no obvious residual tumor
3	45F	Headache	Retrorectalneuroendocrine carcinoma	Stage IIIa (T2N1M0)	Sphenoid sinus, pituitary gland	4m	N	N	Debulking surgery + CT (cisplatin+ etoposide)	6m	Dead
4	47M	Epistaxis	Thyroidpoorly differentiated carcinoma	Stage IVb (pT3N1bM1)	Maxillary, ethmoid, sphenoid sinuses, nasal cavity, orbit, brain	6m	Left iliac	Left iliac	Tumor biopsy + RT + iodine ablation therapy	3y11m	Dead
5	48F	Nasal mass	Thyroid papillary carcinoma	Stage IVc (M1)	Nasal septum	Synchronous	N	N	Tumor excision	2y11m	Alive, no obvious residual tumor
6	49M	Diplopia	Gastricadenocarcinoma	Stage Ib (T1N1M0)	Sphenoid sinus, cavernous sinus	6y3m	N	N	Tumor excision + CT (Capecitabine+ oxaliplatin+ Paclitaxel)+ Target therapy (Cetuximab+ Uracil-Tegafur)	1y4m	Dead
7	51F	EOM limitation, hearing impairment	Breast invasive ductal carcinoma	Stage IV (pT3N3M1)	Ethmoid sinus, nasal cavity, nasal septum	11y	Bone, pleural, lung	Bone, pleural, lung	Tumor biopsy + CT	1m22d	Dead
8	53F	Facial pain, headache, ptosis	Rectal adenocarcinoma	Stage IVb (M1)	Nasal cavity, skull base,	1y1m	Lung, liver, adrenal, bone	Lung, liver, adrenal, bone	Tumor biopsy + CT (Irinotecan + Fluorouracil + Leucovorin) + Target therapy (Bevacizumab + Cetuximab)	9m	Alive with residual tumor
9	56M	Epistaxis, nasal obstruction	Hepatocellular carcinoma	StageIVb (M1)	Frontal, ethmoid sinuses, nasal septum, nasal cavity, orbit	10y	N	Lung, brain	Tumor biopsy + RT+ Target therapy (Sorafenib)	2m	Dead
10	57F	Epistaxis	Lungadenocarcinoma	Stage IVa (cT3N2M1b)	Nasal cavity	Synchronous	Lung, bone	Lung, bone	Tumor biopsy + RT + CT (Alimta + Cisplatin + Docetaxel) + Target therapy (Erlotinib + Pembrolizumab)	11m	Dead
11	66F	Nasal mass Epistaxis	Breast invasive ductal carcinoma	Stage IV (pT4cN0M1)	Nasal vestibule	4y3m	Lung	Lung	Tumor biopsy + Hormone therapy (Tamoxifen)	2y2m	Alive with residual tumor
12	66F	No	Broad ligamentleiomyosarcoma	Stage IV (M1)	ITF	18y7m	N	Liver, abdomen, muscle, bone	Tumor biopsy + CT (Gemcitabine + Taxotere + Cisplatin + Everolimus + Adriamycin) + Target therapy (Pazopanib)	3m	Alive with residual tumor
13	67M	Epistaxis	Right plantar foot melanoma	Stage IIIb (T4aN2bM0)	Maxillary sinus	6 m	N	N	Tumor biopsy + CT (Dacarbazine + Cisplatin + Vinblastine + Proleukin + Cyclophosphamide + paclitaxel) + interferon-A + immunotherapy (IL-2)	1y1m	Dead
14	69M	Nasal mass	Hepatocellular carcinoma	StageIVb (M1)	Nasal cavity	2m	Lung	Lung	Tumor biopsy + CT (Etoposide + Doxorubicin + Cisplatin +5-FU +Leucovorin)	3d	Dead
15	70M	Epistaxis	Cecaladenocarcinoma	Stage IVc (M1)	Maxilla	Synchronous	N	N	Tumor resection + CT (fluorouracil + oxaliplatin + calcium folinate + irinotecan) + Target therapy (bevacizumab)	3m	Dead
16	77M	Nasal obstruction	Supraglottic mucoepidermoid carcinoma	Stage IVa (pT4N2cM0)	Maxillary sinus, nasal cavity, nasal septum	6m	N	N	Tumor resection + RT	2m	Alive, no obvious residual tumor
17	80M	NA	Hepatocellular carcinoma	Stage IVb (M1)	nasal septum	5y9m	N	Lung, muscle, bone	Tumor biopsy	10m	Dead

Abbreviations: F: female; M: male; EOM: extraocular movement; NA: not available; y: year; m: months; d: days; PPF: pterygoid palatine fossa; ITF: infratemporal fossa; RT: radiotherapy; CT: chemotherapy; CCRT: concurrent chemoradiotherapy.

**Table 4:** Review of major case series (cases ≥ 3) of sinonasal metastatic cancers in the English language literature.

Years of Publication	Authors	Total Patients	Age (mean, y/o)	Sex	Metastatic Site	Primary Tumor Sites	Follow-up (months)	Alive with Residual Tumor (grossly)	Death
1959	Garrett [49]	6	58.8	5M1F	5M, 1E	1B, 1G, 1L, 1K, 2T	0.3–16	1	4
1966	Bernstein et al. [5]	10	54.2	3M7F	5M, 1E, 2F, 2N	1B, 2G, 5K, 1L, 1U	1–48	4	5
1987	Som et al. [50]	6	54	NA	3M, 2S, 1 E,1F	6K	NA	0	3
1990	Mickel et al. [12]	7	49.4	5M2F	7S	3L, 1U, 2O, 1 humerus	0.25–7	NA	NA
2000	Simo et al. [22]	6	67.8	3M3F	NA	6K	6–48	2	4
2008	Huang et al. [26]	17	50.8	9M8F	7M, 7N, 3E, 1S, 1NP	5G, 3H, 3K, 3B, 2T, 1L	NA	0	7
2008	Kaminski et al. [23]	4	61	1M3F	2M,1S,1N	1B, 2G, 1K	2–23	0	3
2011	Azarpira et al. [6]	3	58	2M1F	1E, 1M, 1S	1B, 1K, 1U	6–11	0	3
2011	Choong et al. [21]	4	55.8	3M1F	4N	4K	8–24	1	3
2012	Parida et al. [24]	3	54.3	1M2F	2F, 1N	3K	4–6	1	0
2016	Ravnik et al. [25]	3	57	1M2F	3P	1B, 1K, 1 lymphoma	8–48	2	0
2019	Chang et al. (our study)	17	56.8	9M8F	8N, 6SB, 5 septum, 4M, 4E, 4S, 1F,	2B, 4G, 3H, 1L, 2T, 1 supraglottis, 2 leg, 1 retroperitoneum, 1 broad ligament	2–228	7	9
**Total**		86	55.5	42M38F	27M, 11E, 16S, 4F, 23N, 1 NP, 6 SB, 5 septum	10B, 14G, 6H, 7L, 31K, 6T, 3U, 1 supraglottis, 3 extremities, 1 retroperitoneum, 1 broad ligament	0.3–228	18	41

NA: not available; M: maxillary; S: sphenoid; E: ethmoid; F: frontal; N: nasal cavity; P: pituitary gland; NP: nasopharynx; SB: skull base; B: breast; G: gastrointestinal tract; H: hepatic region; K: kidney; L: lung; T: thyroid; U: urogenital region; O: oral.

## Supplementary Materials

The following are available online at https://www.mdpi.com/2077-0383/8/4/539/s1, Figure S1: Survival curves for patients with sinonasal metastasis analyzed based on several parameters.
