# Supplementary figures and images for: Metastatic Tumors of the Sinonasal Cavity: A 15-Year Review of 17 Cases
Source: J Clin Med. 2019 Apr 19;8(4):539. doi: 10.3390/jcm8040539 (PMC6517969; doi:10.3390/jcm8040539)

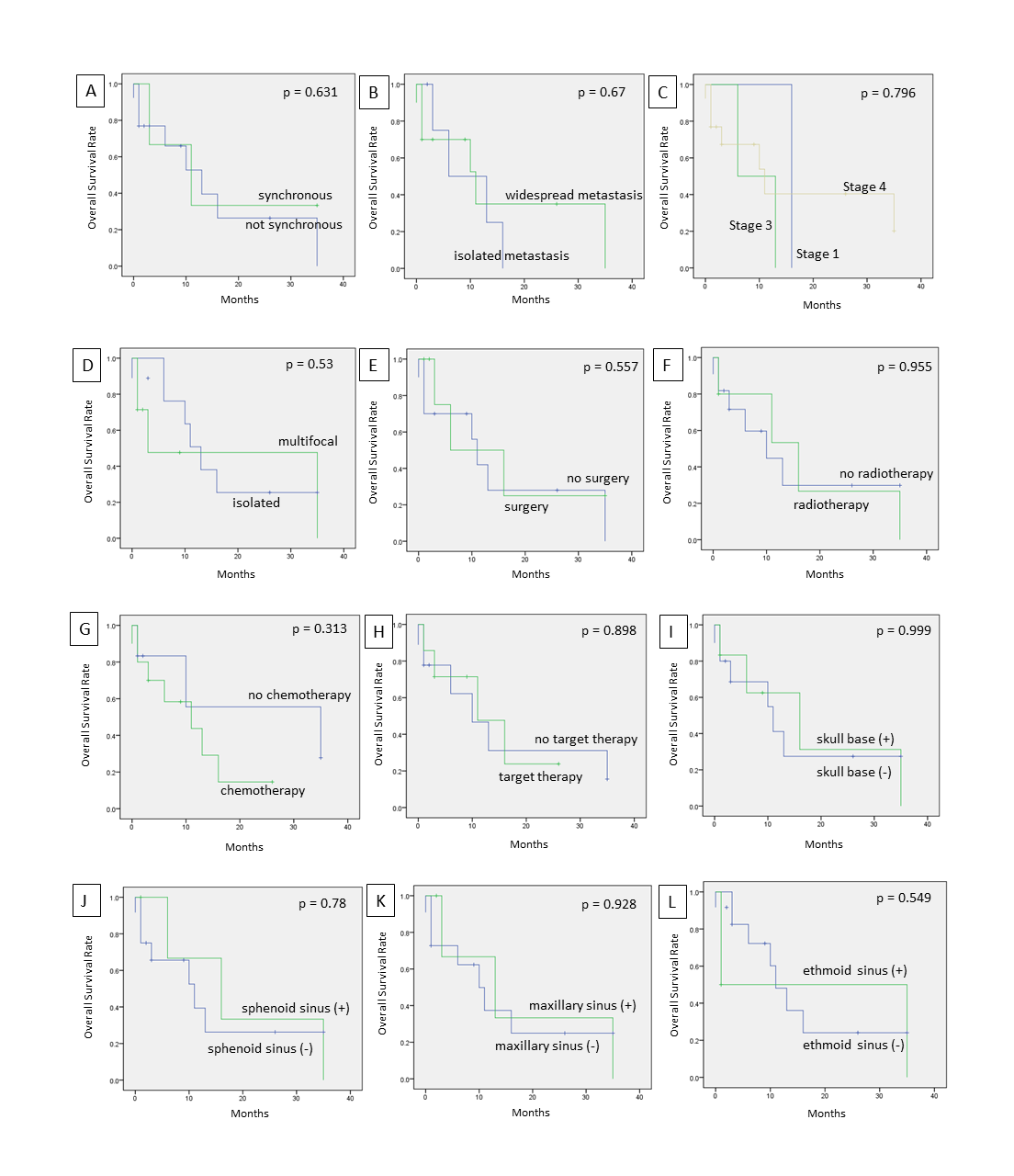

Supplement: Supplementary file 1 [file jcm-08-00539-s001.tif]
